# Supplementary figures and images for: The TIR-domain containing effectors BtpA and BtpB from Brucella abortus impact NAD metabolism
Source: PLoS Pathog. 2020 Apr 16;16(4):e1007979. doi: 10.1371/journal.ppat.1007979 (PMC7188309; doi:10.1371/journal.ppat.1007979)

**A**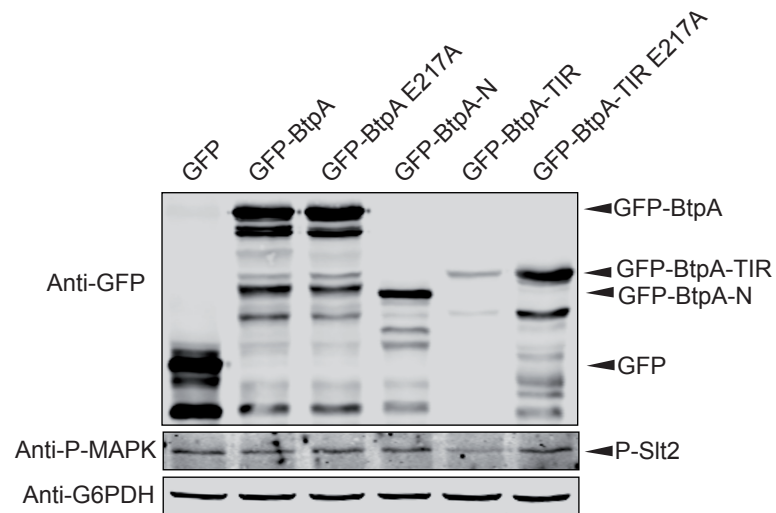**B**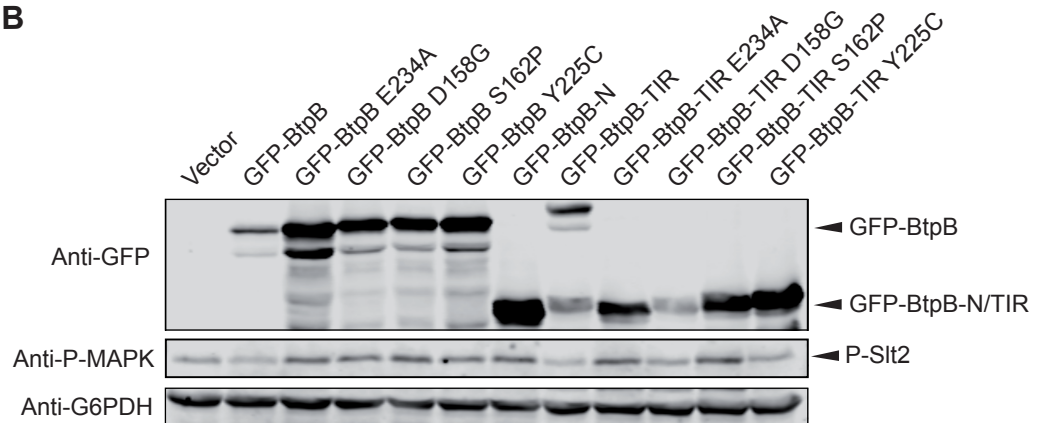**C**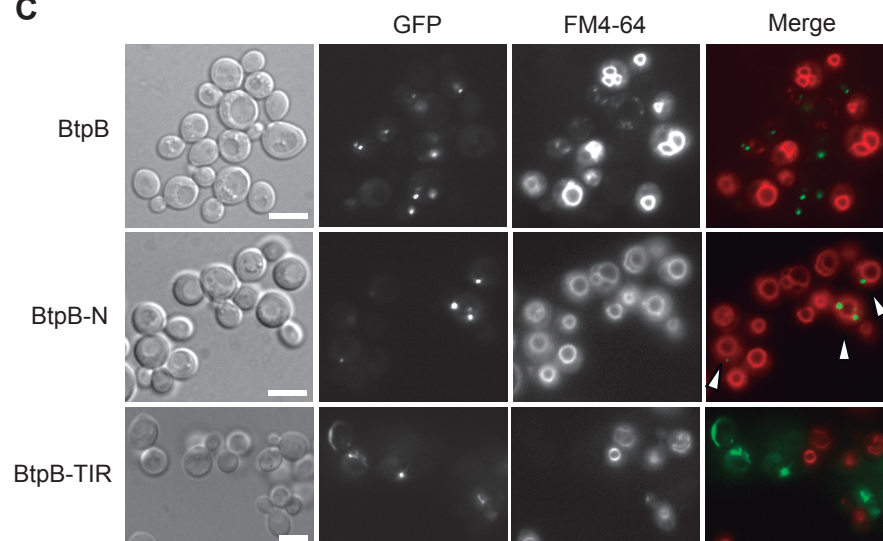

Supplement: S1 Fig — Western blotting of YPH499 cells extracts bearing the indicated BtpA (A) and BtpB (B) versions from pYES2-GFP plasmid derivatives, using antibodies anti-GFP (upper panels), anti-P-MAPK to show dual phosphorylation of Slt2 yeast MAPK and anti-G6PDH as loading control (lower panels). (C) Normarski and fluorescence microscopy of YPH499 cells expressing GFP-BtpB, GFP-BtpB-N and GFP-BtpB-TIR after 4h induction, stained with the endocytic marker FM4-64 for 1h. Scale bars indicate 5 μm. (PDF) [file ppat.1007979.s001.pdf]

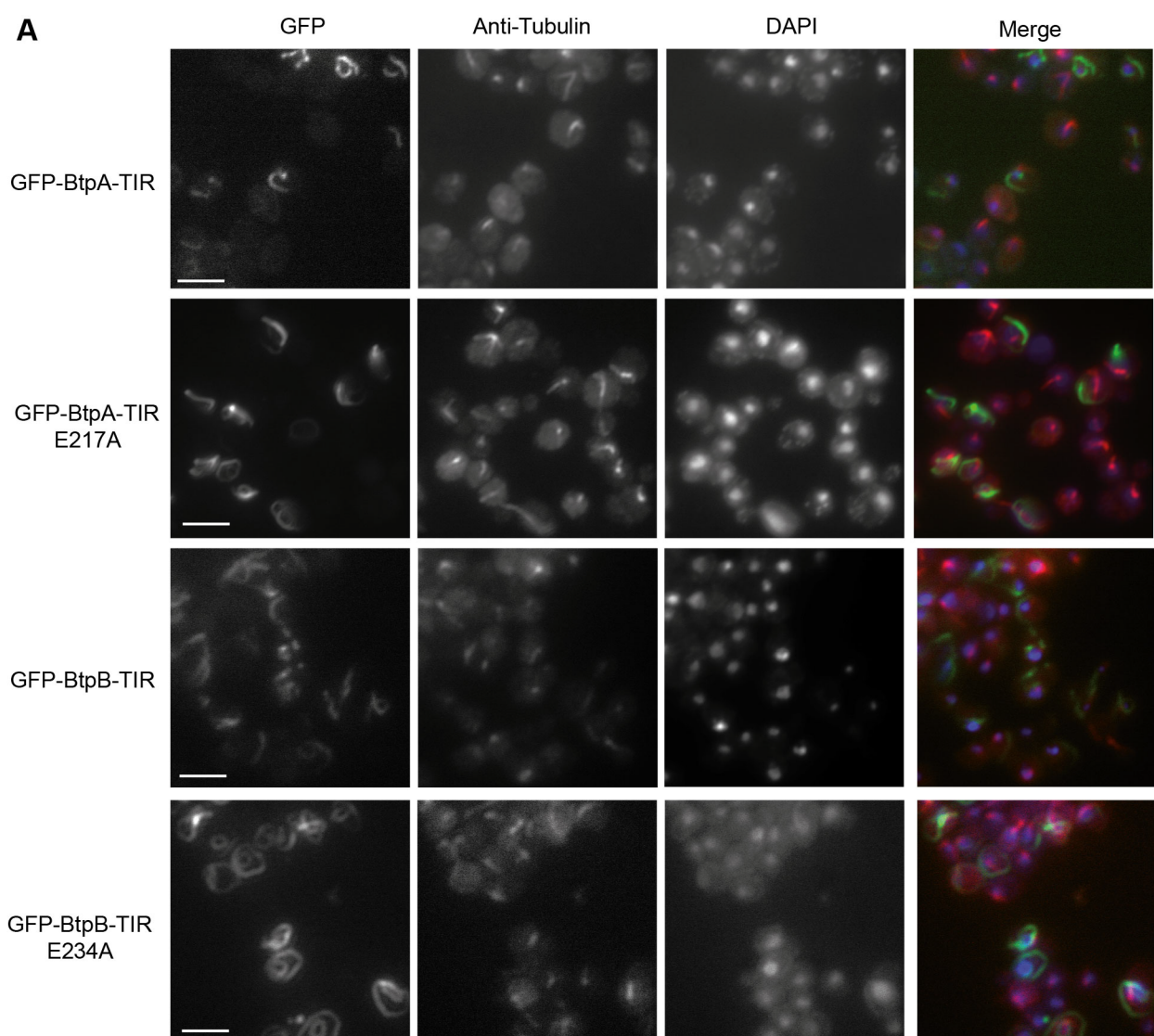

S2 Fig

Supplement: S2 Fig — Indirect immunofluorescence of YPH499 yeast cells expressing GFP-BtpA-TIR, GFP-BtpB-TIR, and their corresponding E234A mutant versions from pYES2 plasmid derivatives (green). Microtubules are stained using anti-tubulin antibody (red). Nuclei are labelled with DAPI (blue). Scale bars correspond to 5 μm. (PDF) [file ppat.1007979.s002.pdf]

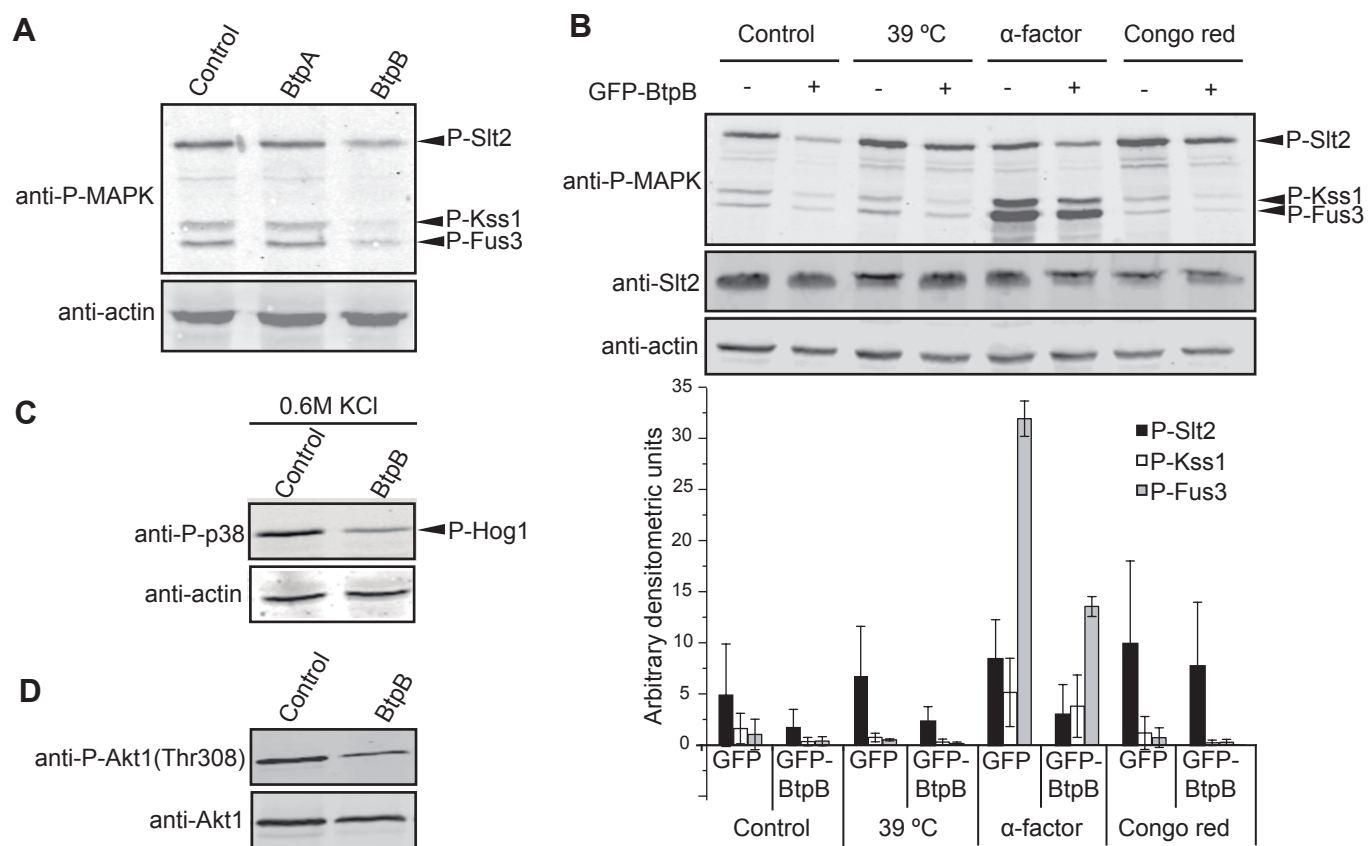

S3 Fig

Supplement: S3 Fig — (A) Western blotting from cells bearing the empty vector pYES2 (control), BtpA or BtpB from pYES2-GFP plasmid derivatives, developed with anti-P-MAPK antibody to detect dually-phosphorylated Slt2, Kss1 and Fus3 (upper panel) and anti-actin to detect actin as loading control. (B) Upper part: representative immunoblot from yeast cell lysates bearing pYES2-GFP-BtpB (+) or pYES2 (-) and upon different conditions: 30ºC (control), high temperature (39ºC), pheromone (α-factor) or Congo red, using anti-P-MAPK (upper panel), anti-Slt2 (medium panel) and anti-actin (lower panel). Lower part: densitometric measurement of WB bands corresponding to phosphorylated MAPKs Slt2, Kss1 and Fus3. The graph displays densitometric data of phosphorylated MAPKs normalized against actin and error bars show the standard deviation from three independent experiments on different transformant clones. (C) Western blotting of cells containing the pYES2 empty vector (control) or pYES2-GFP-BtpB, developed with anti-P-p38 antibody to detect MAPK Hog1 under high osmolarity. conditions (0.6M KCl). (D) Western blotting of cells expressing heterologous Akt1 (pYES3-GFP-Akt1) with either pYES2 empty vector (control) or pYES2-GFP-BtpB, using anti-P-Akt1(Thr)308 (upper panel) and anti-Akt1 antibodies. All immunoblots were performed on protein extracts from transformants of the YPH499 yeast strain after 4 h of galactose induction. (PDF) [file ppat.1007979.s003.pdf]

**A**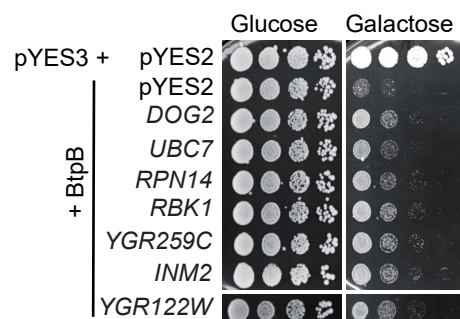**B**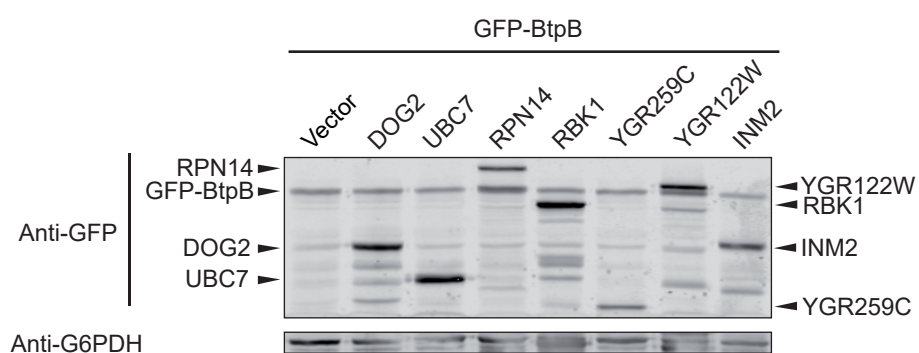**C**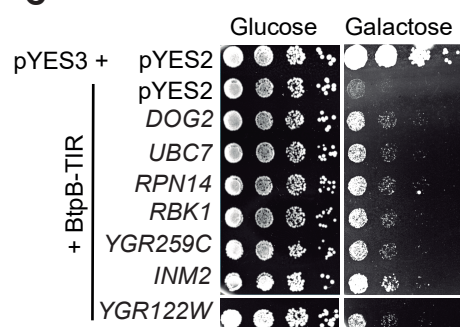**D**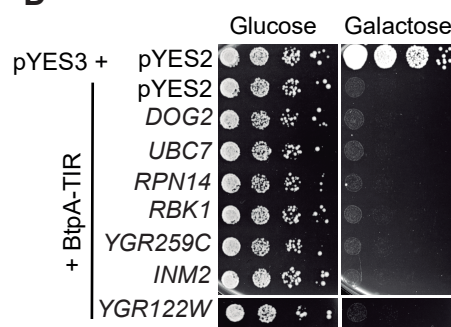

S4 Fig

Supplement: S4 Fig — (A) Ten-fold serial dilution assay of yeast cells co-expressing BtpB and each of the seven suppressor ORFs isolated from a yeast genetic screen. pYES3 and pYES2 are the corresponding empty vectors for BtpB and for the overexpressed genes, respectively. (B) Western blotting of W303-1A yeast strain co-expressing GFP-BtpB and each of the proteins encoded by the suppressor genes. Antibodies anti-GFP to detect GFP-BtpB (upper panel) and Anti-G6PDH as loading control (lower panel) were used. Anti-GFP antibody allows the detection of the indicated protein A-tagged proteins due to affinity of the tag with the Fc region of IgG-type antibodies. (C) and (D) Ten-fold serial dilution assays of yeast cells co-expressing BtpB-TIR (C) or BtpA-TIR (D) and the suppressor genes. pYES3 and pYES2 are the corresponding empty vectors for BtpB- or BtpA-TIR and for the overexpressed genes, respectively. (PDF) [file ppat.1007979.s004.pdf]

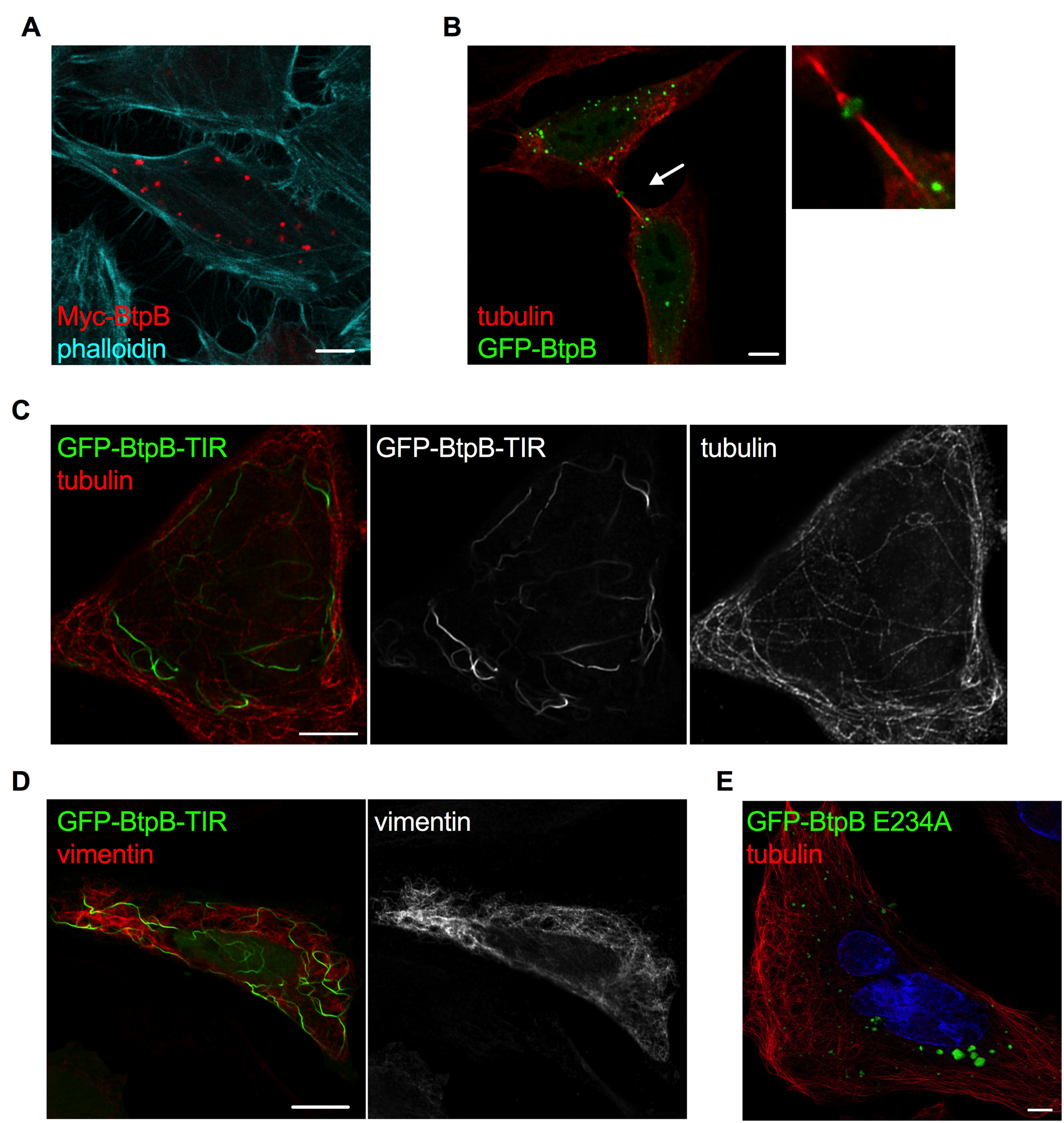

Supplement: S6 Fig — (A) Representative micrograph of HeLa cells expressing Myc-BtpB revealed with an anti-Myc antibody (red) and phalloidin for labelling actin (cyan). (B) GFP-BtpB (green) can be detected at intercellular contacts (arrow and zoomed image). Cells were labeled with an anti-tubulin antibody (red). (C) HeLa cells were transfected with GFP-BtpB-TIR (green) and then labelled for tubulin (red) or (D) vimentin (red). (E) Representative image of cells labelled with anti-tubulin antibody (red) expressing aggregates of GFP-BtpB E234A (green). Scale bars correspond to 5 μm. (TIF) [file ppat.1007979.s006.tif]

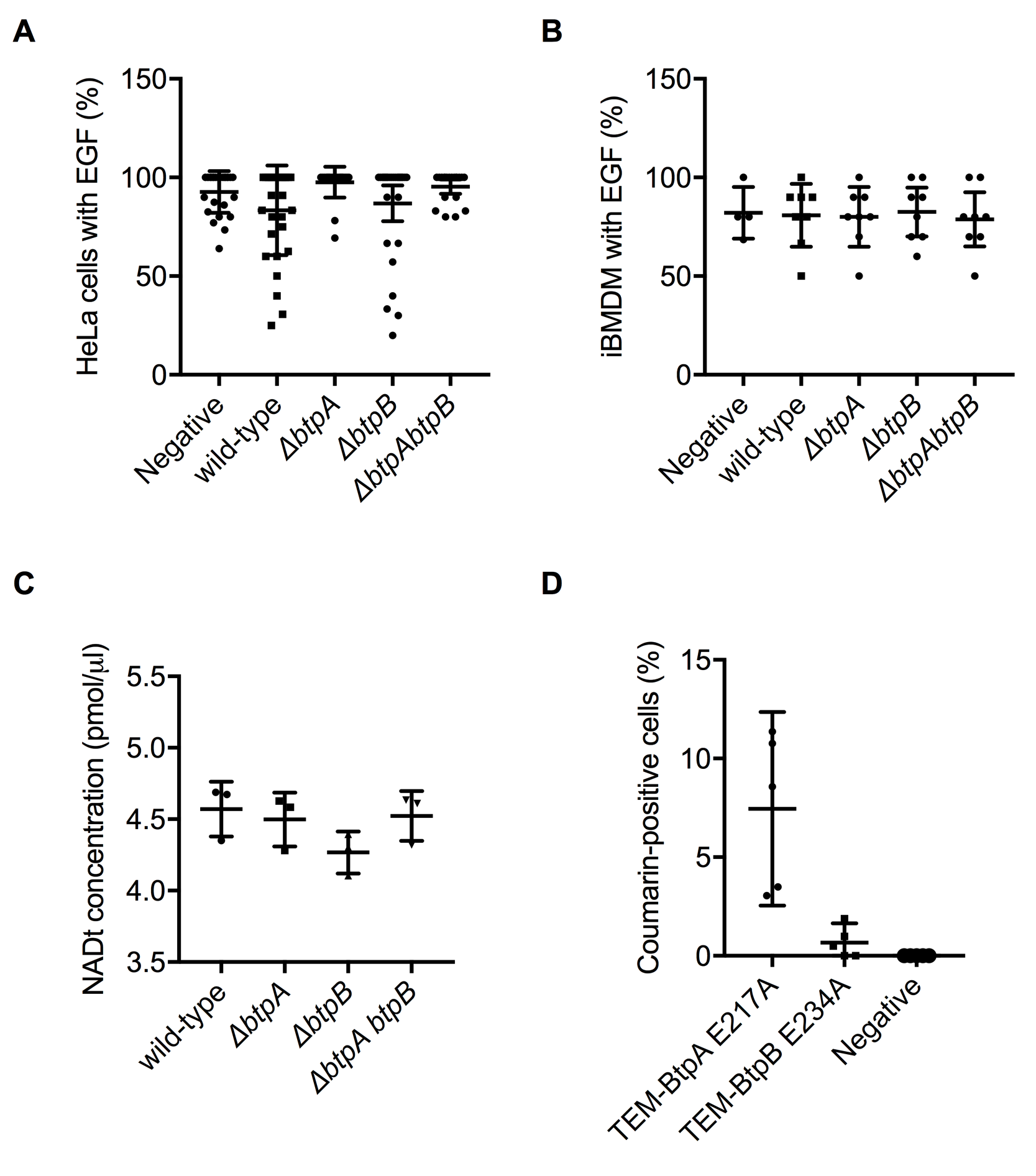

Supplement: S7 Fig — (A) HeLa cells or (B) iBMDM infected with either wild-type or a mutant strain lacking btpA, btpB or both genes were incubated with EGF conjugated with Alexa Fluor 555 for 10 minutes and the percentage of infected cells showing uptake of this fluorescent marker quantified by microscopy at 24h post-infection. Counts correspond to individual microscopy fields, obtained from three independent experiments. Mock infected cells are included as a control (negative). Data correspond to means ± standard deviation and statistical comparison was done with one-way ANOVA test, with no statistical significance observed. (C) Control total NAD levels from the inocula, corresponding to bacterial cultures of wild-type B. abortus, or strains lacking btpA, btpB or both btpAbtpB. Data correspond to means ± standard deviation from three independent experiments and statistical comparison was done with Kruskal-Wallis test, with no statistical significance observed. (D) RAW macrophages were infected with wild-type B. abortus carrying N-terminal TEM-1 fused BtpA E217A or BtpB E234A catalytic mutants for 24 h. Data represents the means ± standard errors of the percentage of cells with coumarin fluorescence from 5 independent experiments, in which at least 100 cells were analyzed per experiment and condition. (TIF) [file ppat.1007979.s007.tif]
