# Supplementary material for: The TIR-domain containing effectors BtpA and BtpB from Brucella abortus impact NAD metabolism
Source: PLoS Pathog. 2020 Apr 16;16(4):e1007979. doi: 10.1371/journal.ppat.1007979 (PMC7188309; doi:10.1371/journal.ppat.1007979)
Supplement: S5 Fig — (A) Alignment of protein sequences of the TIR domains of BtpB, BtpA, human SARM1 and plant RUN1. Conserved residues relevant for this study are marked with the same color code as in Fig 4, except for for the catalytic site residues W213 and E217, that are colored in pink. (B) Structure of BtpA-TIR (left; PDB: 4LZP)) and RUN1-NADP+ complex (right; PDB: 6O0W), showing the equivalent positions of residues mutated in BtpB isolated in the yeast screen. Both structures cartoons are displayed in the same orientation. Side chain of mutated residues of BtpA relevant for this study are colored as in (A). The side chains of residues of the catalytic site of RUN1 are shown as ball-and-sticks and colored in pink and the NADP+ ligand is colored in cyan. Specific atoms are colored as follows: nitrogen in blue, oxygen in red and phosphorus in orange. (C) Phenotype of selected loss-of-function BtpB mutants. Ten-fold serial dilution growth assay of YPH499 cells transformed with pYES2 empty vector and pYES2 plasmid derivatives expressing full-length BtpB wild-type and mutants D158G, S162P and Y225C, under control (Glucose) and induction (Galactose) conditions. (D) Nomarski and fluorescence microscopy images of YPH499 cells expressing the GFP-BtpB indicated mutants, after 4h induction, stained with the endocytic marker FM4-64 for 1h. Scale bars indicate 5 μm. (E) Graph from the same experiment as in C representing the percentage of cells showing both GFP and FM4-64 vacuolar signal. Results correspond to means ± standard deviation of three independent transformants (n ≥ 100) and statistical comparison was done with one-way ANOVA with a p-value < 0.0001 (***) for all four mutants versus wild-type. (PDF) [file ppat.1007979.s005.pdf]

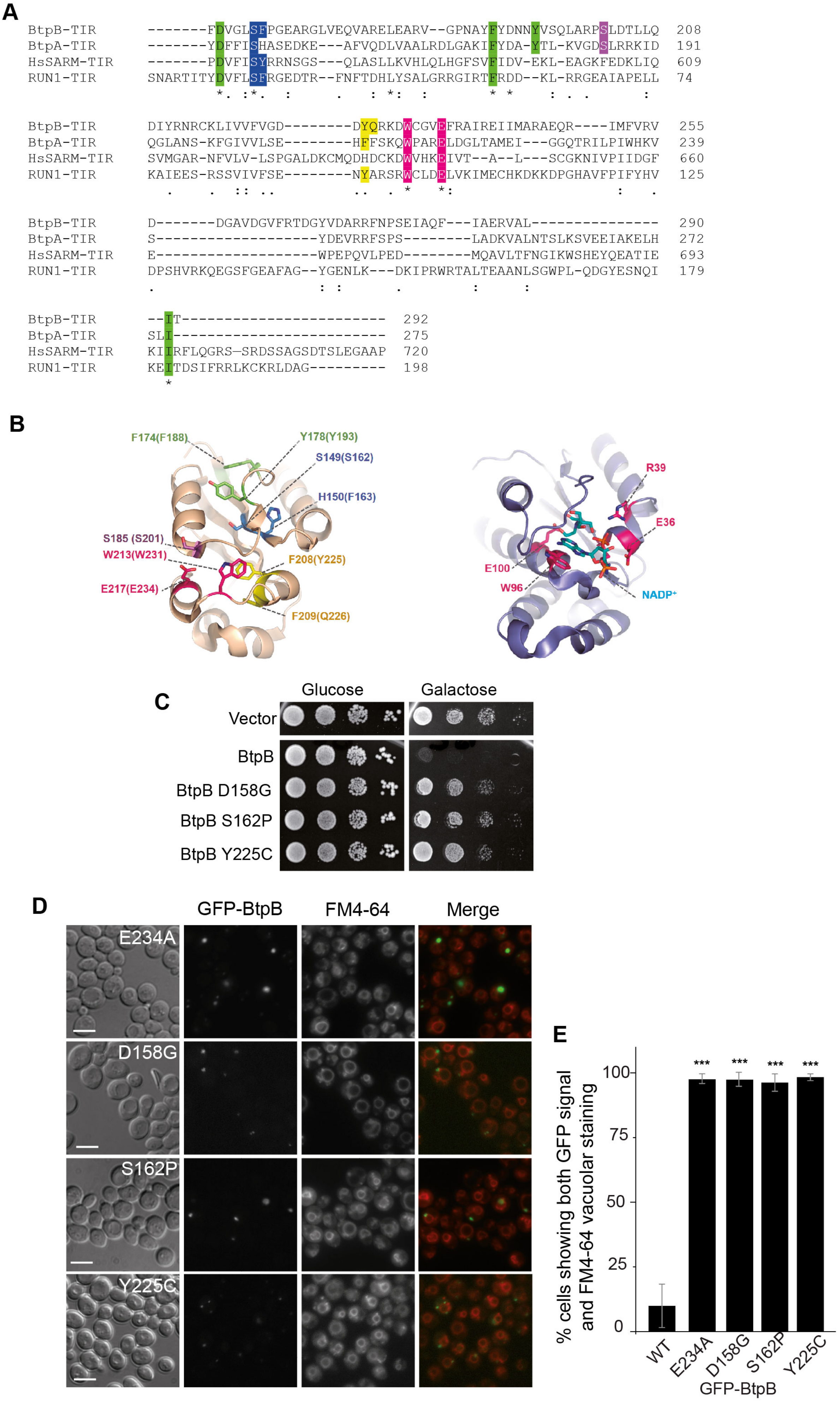
A
BtpB-TIR -------FDVGLSFPGEARGLVEQVARELEARV--GPNAYFYDNNYVSQLARPSLDTLLQ 208
BtpA-TIR -------YDFFISHASEDKE---AFVQDLVAALRDLGAKIFYDA-YTL-KVGDSLRRKID 191
HsSARM-TIR -------PDVFISYRRNSGS---QLASLLKVHLQLHGFSVFIDV-EKL-EAGKFEDKLIQ 609
RUN1-TIR SNARTITYDVFLSFRGEDTR--FNFTDHLYSALGRRGIRTFRDD--KL-RRGEAIAPELL 74
BtpB-TIR DIYRNRCKLIVVFVGD--------DYQRKDWCGVEFRAIREIIMARAEQR----IMFVRV 255
BtpA-TIR QGLANS-KFGIVVLSE--------HFFSKQWPAREELDGLTAMEI---GGQTRILPIWHKV 239
HsSARM-TIR SVMGAR-NFVLV-LSPGALDKCMQDHDCKDWVHKEIVT--A--L---SCGKNIVPIIDGF 660
RUN1-TIR KAIEES-RSSVIVFSE--------NYARSRWCLDELVKIMECHKDKKDPGHAVFPIFYHV 125
BtpB-TIR D-------DGAVDGVFRTDGYVDARRFNPSEIAQF---IAERVAL-------------- 290
BtpA-TIR S------------------YDEVRRFSPS--------LADKVALNTSLKSVEEIAKELH 272
HsSARM-TIR E------------------WPEPQVLPED--------MQAVLTFNGIKWSHEYQEATIE 693
RUN1-TIR DPSHVRKQEGSFGEAFAG--YGENLK---DKIPRWRTALTEAANLSGWPL-QDGYESNQI 179
BtpB-TIR --IT----------------------- 292
BtpA-TIR SLI------------------------ 275
HsSARM-TIR KIIRFLQGRS—SRDSSAGSDTSLEGAAP 720
RUN1-TIR KEITDSIFRRLKCKRLDAG--------- 198
B
F174(F188)
Y178(Y193)
S149(S162)
H150(F163)
S185 (S201)
W213(W231)
F208(Y225)
E217(E234)
F209(Q226)
R39
E36
E100
W96
NADP+
C
Glucose
Galactose
Vector
BtpB
BtpB D158G
BtpB S162P
BtpB Y225C
D
GFP-BtpB
FM4-64
Merge
E234A
D158G
S162P
Y225C
E
% cells showing both GFP signal and FM4-64 vacuolar staining
100
75
50
25
0
***
WT
E234A
D158G
S162P
Y225C
GFP-BtpB

S5 Fig
