# Supplementary material for: The TIR-domain containing effectors BtpA and BtpB from Brucella abortus impact NAD metabolism
Source: PLoS Pathog. 2020 Apr 16;16(4):e1007979. doi: 10.1371/journal.ppat.1007979 (PMC7188309; doi:10.1371/journal.ppat.1007979)
Supplement: S1 Table — (DOCX) [file ppat.1007979.s008.docx]

Table S1. Plasmids generated and used in this work

| Plasmid | Description | Source/Refererence |
| --- | --- | --- |
| pYES2 | Control *URA3*-based yeast empty vector | Invitrogen |
| pYES2-GFP | *URA3*-based yeast expression episomal plasmid for N-terminal GFP fusions, under *GAL1* promoter. | [1] |
| pGEM-T-easy-BtpA | PCR template of *B. abortus* *btpA*. | [2] |
| pGEM-T-easy-BtpB | PCR template of *B. abortus btpB*. | [3] |
| pYES2-GFP-BtpA | *Brucella* BtpA full length GFP fusion for yeast expression, under *GAL1* promoter. | This work |
| pYES2-GFP-BtpB | *Brucella* BtpB full length GFP fusion for yeast expression, under *GAL1* promoter. | This work |
| pYES3-GFP | *TRP1*-based yeast expression episomal plasmid for N-terminal GFP fusions, under *GAL1* promoter | This work |
| pYES3-GFP-BtpB | *Brucella* BtpB full length GFP fusion for yeast expression, in a *TRP1*-based plasmid, under *GAL1* promoter. | This work |
| pYES2-GFP-BtpA-N | N-terminal (1-126), non-TIR domain of BtpA´s GFP fusion for yeast expression, under GAL1 promoter. | This work |
| pYES2-GFP-BtpA-TIR | C-terminal (127-275), TIR domain of BtpA´s GFP fusion for yeast expression, under GAL1 promoter. | This work |
| pYES3-GFP-BtpA-TIR | C-terminal (127-275), TIR domain of BtpA´s GFP fusion for yeast expression, in a *TRP1*-based plasmid, under *GAL1* promoter. | This work |
| pYES2-GFP-BtpB-N | N-terminal (1-139), non-TIR domain of BtpB´s GFP fusion for yeast expression, under GAL1 promoter. | This work |
| pYES2-GFP-BtpB-TIR | C-terminal (140-292), TIR domain of BtpB´s GFP fusion for yeast expression, under GAL1 promoter. | This work |
| pYES3-GFP-BtpB-TIR | C-terminal (140-292), TIR domain of BtpB´s GFP fusion for yeast expression, in a *TRP1*-based plasmid, under *GAL1* promoter. | This work |
| pYES2-GFP-BtpA E217A | Catalytic inactive mutant full length BtpA GFP fusion for yeast expression. | This work |
| pYES2-GFP-BtpA-TIR E217A | Catalytic inactive mutant TIR domain BtpA GFP fusion for yeast expression. | This work |
| pYES2-GFP-BtpB E234A | Catalytic inactive mutant full length BtpB GFP fusion for yeast expression. | This work |
| pYES3-GFP-BtpB E234A | The same but in a different yeast auxotrophic marker (*TRP1*). | This work |
| pYES2-GFP-BtpB-TIR E234A | Catalytic inactive mutant TIR domain BtpB GFP fusion for yeast expression. | This work |
| pYES2-GFP-BtpB-TIR D158G | Loss-of-function mutant TIR domain BtpB GFP fusion for yeast expression. | This work |
| pYES2-GFP-BtpB-TIR S162P | Loss-of-function mutant TIR domain BtpB GFP fusion for yeast expression. | This work |
| pYES2-GFP-BtpB-TIR Y225C | Loss-of-function mutant TIR domain BtpB GFP fusion for yeast expression. | This work |
| pYES3-GFP-Akt1 | Yeast plasmid for heterologous Akt1expression | [4] |
| YCpLG-PI3Kα-CAAX | Yeast plasmid for heterologous PI3K expression | [1] |
| pBBR-BtpA | Complementing vector expressing BtpA | This work |
| pBBR-BtpB | Complementing vector expressing BtpB | This work |
| pBBR-BtpA E217A | Complementing vector expressing catalytic mutant of BtpA | This work |
| pBBR-BtpB E234A | Complementing vector expressing catalytic mutant of BtpB | This work |
| pTEM-BtpA E217A | TEM1 lactamase fusion with BtpA catalytic mutant | This work |
| pTEM-BtpB 234A | TEM1 lactamase fusion with BtpB catalytic mutant | This work |
