# Supplementary material for: The TIR-domain containing effectors BtpA and BtpB from Brucella abortus impact NAD metabolism
Source: PLoS Pathog. 2020 Apr 16;16(4):e1007979. doi: 10.1371/journal.ppat.1007979 (PMC7188309; doi:10.1371/journal.ppat.1007979)
Supplement: S2 Table — (DOCX) [file ppat.1007979.s009.docx]

Table S2. Oligonucleotides used in this work.

| Name | Sequence |
| --- | --- |
| BtpA-UP | (5’-cgcggatccatgagttcgtactcttctaata-3’) |
| BtpA-LO | (5’-ggaattctcagataagggaatgcagttc-3’) |
| BtpB-UP | (5’-cgcgggatccatgtacaatttatttgtttcggg-3’) |
| BtpB-LO | (5’-gctctagactaggtgatgagggcgacg-3’) |
| BtpB-UP-pYES3 | (5’-cgggatccatgtacaatttatttgtttcgggc-3’) |
| BtpB-LO-pYES3 | (5’-cgggatccctaggtgatgagggcgac-3’) |
| BtpA-126stop-LO | (5’-ggaattctcatgattgtttgatgaaagcttca-3’) |
| BtpA-BamHI127 UP | (5’-cgggatccttgagctccatgcgaacaac-3’) |
| BtpB-140stop-LO | (5’-ggaattcctacagagttattcct-3’) |
| BtpB-BamHI140 UP | (5’-cgggatccatgccgtcgtggacgcgacag-3’) |
| BtpB-EcoRI-LO | (5’-ggaattcctaggtgatgagggcgacg-3’) |
| Mut-BtpAE217A-UP | (5’-agcaatggcccgcaagagcattagatggactgacggc-3’) |
| Mut-BtpAE217A-LO | (5’-gccgtcagtccatctaatgctcttgcgggccattgct -3’) |
| Mut-BtpBE234A-UP | (5’-aaaagactggtgcggcgtcgcgttccgcgcgattcgcgaa-3’) |
| Mut-BtpBE234A-LO | (5’-ttcgcgaatcgcgcggaacgcgacgccgcaccagtctttt-3’) |
| Mut-BtpBD158G-UP | (5’-cacgattttggcgtcggtctctcttttccc-3’) |
| Mut-BtpBD158G-LO | (5’-gagaccgacgccaaaatcgtgctgagtgata-3’) |
| Mut-BtpBS162P-UP | (5’-cgtcggtctcccttttcccggtgag-3’) |
| Mut-BtpBS162P-LO | (5’-ccgggaaaagggagaccgacgtcaaaatc-3’) |
| Mut-BtpBY225C-UP | (5’-ggcgacgactgtcagcgaaaagactggt-3’) |
| Mut-BtpBY225C-LO | (5’-ttttcgctgacagtcgtcgccgacgaaga-3’) |
| mutazarUP | (5’-cgcgccttcccacgattttc-3’) |
| mutazarLO | (5’-gcaaggttttcagtataatg-3’) |
| BtpB Fw | (5’-ggggacaagtttgtacaaaaaagcaggcttcatgtacaatttatttgtttcg-3’) |
| BtpB Rv | (5’-ggggaccactttgtacaagaaagctgggtcctaggtgatgagggcgacgc-3’) |
| BtpB-TIR Fw | (5’-ggggacaagtttgtacaaaaaagcaggcttcatgccgtcgtggacgcgacaggc-3’) |
| BtpB-N Rv | (5’-ggggaccactttgtacaagaaagctgggtcctacagagttattcctttggccg-3’) |
| BtpA Fw-pBBR-1-MCS4 | (5’-aaaaaactagtatgagttcgtactcttctaata-3’) |
| BtpA Rv-pBBR-1-MCS4 | (5‘-aaaaagaattctcagataagggaatgcagttc-3’) |
| BtpB Fw-pBBR-1-MCS4 | (5’-aaaaagagctcatgacatctagtcgcgacac-3’) |
| BtpB Rv-pBBR-1-MCS4 | (5’-aaaaaactagtctaggtgatgagggcgacg-3’) |
| BtpA_E217A_ Fw | (5’-ccgcaagagccttagatggactgacggca-3’) |
| BtpA_E217A_ Rv | (5’-catctaaggctcttgcgggccattg-3’) |
| BtpB_E234A_ Fw | (5’-ggcgtcgccttccgcgcgattcgc-3’) |
| BtpB_E234A_ Rv | (5’-cggaaggcgacgccgcaccagtc-3’) |
| TEM-BtpA_E217A_ FW-pFlagTEM | (5’-ggtgcctcactgattaagcattggtctagaatgagttcgtactcttctaa-3’) |
| TEM-BtpA_E217A_ Rv-pFlagTEM | (5’-ccagtgtgctggaattcgcccttactgcagtcagataagggaatgcagttc-3’) |
| TEM-BtpB_E234A_ FW-pFlagTEM | (5’-ggtgcctcactgattaagcattggtctagaatgacatctagtcgcgacacg-3’) |
| TEM-BtpB_E234A_ Rv-pFlagTEM | (5’-ccagtgtgctggaattcgcccttactgcagctaggtgatgagggcgacgc-3’) |
