# Supplementary material for: The TIR-domain containing effectors BtpA and BtpB from Brucella abortus impact NAD metabolism
Source: PLoS Pathog. 2020 Apr 16;16(4):e1007979. doi: 10.1371/journal.ppat.1007979 (PMC7188309; doi:10.1371/journal.ppat.1007979)
Supplement: S3 Table — (DOCX) [file ppat.1007979.s010.docx]

Table S3. Yeast genes that suppress BtpB-induced toxicity when overexpressed.

| Gene Ontology | ORF | Name | Protein function | Times isolated |
| --- | --- | --- | --- | --- |
| Metabolism | *YHR043C* | *DOG2* | 2-deoxyglucose-6-phosphate phosphatase; confers 2-deoxyglucose resistance when overexpressed. | 1 |
|  | *YCR036W* | *RBK1* | Putative ribokinase. | 1 |
|  | *YGR259C* | *YGR259C* | Dubious open reading frame; overlaps almost completely with the verified ORF *TNA1/YGR260W* | 3 |
|  | *YDR287W* | *INM2* | Inositol monophosphatase, involved in biosynthesis of inositol | 1 |
| Ubiquitin-proteasome | *YMR022W* | *UBC7* | Ubiquitin conjugating enzyme; involved in the ER-associated protein degradation (ERAD) pathway and in the inner nuclear membrane-associated degradation (INMAD) pathway. | 3 |
|  | *YGL004C* | *RPN14* | 19S proteasome regulatory particle (RP) assembly-chaperone; putatively involved in the assembly of the proteasome base subcomplex. | 1 |
| pH regulation | *YGR122W* | *YGR122W* | Protein of unknown function, probable ortholog of *Aspergillus nidulans* PalC, which is involved in pH regulation and binds to the ESCRT-III complex. | 1 |
