# Supplementary material for: The TIR-domain containing effectors BtpA and BtpB from Brucella abortus impact NAD metabolism
Source: PLoS Pathog. 2020 Apr 16;16(4):e1007979. doi: 10.1371/journal.ppat.1007979 (PMC7188309; doi:10.1371/journal.ppat.1007979)
Supplement: S4 Table — (DOCX) [file ppat.1007979.s011.docx]

Table S4. BtpB loss-of-function mutants found by random mutagenesis screening on yeast.

| BtpB mutations | Toxicity |
| --- | --- |
| Y225C | None |
| D158G | None |
| D156N +Y187stop | None |
| Q226P | None |
| V260A + R265C | None |
| S201P | None |
| I291T | Partial |
| F163S | None |
| Y193H | None |
| F188L | None |
| D257G | Partial |
| S162P | None |
