# Supplementary material for: The TIR-domain containing effectors BtpA and BtpB from Brucella abortus impact NAD metabolism
Source: PLoS Pathog. 2020 Apr 16;16(4):e1007979. doi: 10.1371/journal.ppat.1007979 (PMC7188309; doi:10.1371/journal.ppat.1007979)
Supplement: Attachment — Submitted filename: Response to the editor and reviewers.pdf [file ppat.1007979.s012.pdf]

Dear Editor and Reviewers,

We would first like to thank all the reviewers for their comments and suggestions that have greatly improved the manuscript. We also thank the editorial team for giving us additional time to address the reviewer's comments. Please find bellow our point-by-point answer. We hope the revised manuscript will be acceptable for publication. All modifications are highlighted in blue in the manuscript.

Editor:

Dear Dr Salcedo,
We are returning your manuscript with three reviews. As you will see, the reviewers all appreciated the amount and quality of the work described in the manuscript, but came to different conclusions about the relevance and significance of the paper, ranging from a "…huge and original work of high interest…" to "…potentially non-specific effects observed with overexpression of the BtpB or the TIR domains of BtpA and/or BtpB…". We tend to agree that the study is heavily weighted towards ectopic expression approaches and requires additional experimentation using infection models to strengthen its conclusions and relevance.
We have carried out complementation assays with full length and catalytic mutant of BtpA and BtpB. The results clearly show that the NAD consuming activity observed in transfection is also occurring during infection (especially in macrophages), validating the ectopic expression data and assigning an important function to these two *Brucella* effectors. To balance the manuscript and decrease the weight of ectopic expression experiments, several figures were moved to supplementary and the data presentation rearranged.

After reading the reviews and looking at the manuscript, we have rendered a decision Major Revision based on the critiques from Reviewers 2 and 3. A significant concern shared by these reviewers that requires your outmost attention is the need to further corroborate the authors' observations made using ectopic expression approaches in yeast and HeLa cells with experimental data generated using infection models, in order to establish the relevance of the BtpA and BtpB-induced phenotypes. In particular, it appears essential to:

1. confirm that the effect of BtpA and BtpB on NAD+ levels during infection depends on their TIR domain NAD hydrolase activity via genetic complementation approaches;
This has been done in both HeLa and macrophage models of infection. In HeLa cells we could nicely complement BtpA and this was dependent on the catalytic NAD consuming activity. The significant variation of the mutant lacking BtpB did not allow us to attain statistical significance with the tests we use (t-tests, most often used in these assays would have yielded significance but they are not appropriate). Due to the extremely high costs of these experiences we did not increase the number of replicates. However, in bone marrow-derived macrophages, most likely due to a higher rate of infected cells, we could clearly establish that BtpA and BtpB catalytic activities are responsible for NAD depletion during infection. These data have now been included in the manuscript (Figure 9 and 10).

2. address the concern raised by Reviewer 3 about the redundant role(s) of BtpA and BtpB on NAD+ levels during infection;
Based on Genetics, lack of additive effect of mutations in two genes can be explained by either a possible interaction between the encoded proteins (i.e., the formation of a complex) or that they are acting on the same pathway/process. Non-additive interactions between two mutations (epistatic) are frequent and can even be modulated depending on the conditions tested, making it difficult to make genetic predictions. However, overall, the lack of additive effect of the *btpA* and *btpB* mutations is indicative of a functional relationship between these two genes. Whether by compensatory effects along a common pathway or direct molecular interactions is yet to be determined.

3. verify that BtpA and BtpB's effects on metabolism (and probably other phenotypes revealed in yeast) during infection is reproducible in macrophages.
The phenotype of NAD reduction was even more striking in macrophages. This data is now included in the manuscript (Figure 10).

We would also like to encourage you to address all the other reviewers' comments in your revised manuscript and responses. We are sorry We cannot be more positive at the moment, however we are looking forward to receiving your revision. Note that we may send your paper back to some of the reviewers upon resubmission.
The majority of all other comments have been addressed by text modifications or by including new data (see response to the reviewers).

Reviewer's Responses to Questions

Part I – Summary

Reviewer #1:

The authors report a new characterization of two Brucella effectors, BtpA and BtpB. They start by showing that these two proteins are toxic for yeast, which depends on their TIR domain. They also report that BtpB generate several defects in yeast, such as a blockage of endocytosis. After screening for suppressors, they realize that NAD+ and ATP levels are affected in yeast producing BtpA or BtpB. They used elegant controls to show that it is indeed the catalytic activity of BtpB, able to degrade NAD+, that is responsible for toxicity in yeast. The authors also show that BtpB is inhibiting endocytosis in humans cells, that BtpA and BtpB can also generate a drop in the level of NAD+ in humans cells, and -importantly- they show that Brucella infection generates a decrease in the NAD+ pool that is not observed in a btpA or a btpB mutant.

This is a huge and an original work of high interest, an interest that is not limited to the Brucella research community. The approach is very interesting, starting from yeast to generate focused and testable hypotheses on human cells. It also generates a new possible function for this family of effectors, suggesting that the pioneering study of Essuma et al. (2017 and 2018) is indeed applicable to several pathogens. This works thus opens new perspectives to the field of host-pathogen interactions, at the molecular and cellular levels.

I have a list of minor issues that should be taken into account for the submission of revised version of this manuscript.

Minor comments have been addressed (see bellow).

Reviewer #2:

This is an interesting paper that suggests the Brucella modulates host metabolic pathways through the activities of two type IV secreted effectors, BtpA and BtpB. The majority of these conclusions are based on potentially non-specific effects observed with overexpression of the BtpB or the TIR domains of BtpA and/or BtpB in yeast and mammalian cells. These phenotypes can be interpreted to be due to the recently identified NAD+ consuming activity of TIR domains- which are found in these effectors. However, it is unclear how the heterologous expression studies would have led the investigators to determine this likely function of the effectors, in the absence of the prior published studies which established BtpA as a NAD+-consuming protein.

Indeed, we took a long time to understand the reason behind the endocytosis blocking during transfection and over-expression in yeast. We first noticed an impact on the ATP levels in yeast before Essuman's paper, and when his work was published, we were quite excited to see that the TIR domains (eukaryotic and prokaryotic) retained enzymatic activity to cleave NAD. We, therefore, built on Essuman's work, which showed this TIR domain NAD-consuming activity by over-expression of different eukaryotic and bacterial TIR domains in *E. coli*. This is the beauty of science; we build on each other's work to slowly understand the role of these proteins during infection, which is ultimately our goal in the case of *Brucella* pathogenesis.

The bulk of the data presented is focused on characterizing the phenotypes observed with the overexpression of these effectors in these cells. Very little is done to actually determine their roles during an infection, including evidence that BtpA and/or BtpB directly regulate energy metabolism during an infection. In addition, no clear connection between the role of the proposed NAD-consuming activity of the TIR domains and their previously identified roles in regulating innate immune responses.

We have extended our infection studies to assign the NAD-consuming activity in both HeLa and macrophages. We did not investigate its link to control of innate immune responses, but this is interesting (and complex!) work that is currently ongoing in the Salcedo lab. We note that the impact on NFkB and cytokine secretion was observed at 24h post-infection, whereas NAD depletion only at 48h, but we are yet to establish if the two are directly connected. We are currently constructing all the tools needed to address these points adequately, and we feel this is beyond the scope of this manuscript.

Reviewer #3:

1. During infection, why do the BtpA and BtpB mutations exert the same effect, completely recovering NAD+? If both are expressed during infection, shouldn't there be only partial recovery or an additive effect in the double mutant?

Not necessarily. Lack of additive effect suggests that *btpA* and *btpB* are epistatic. Based on Genetics, lack of additive effect of mutations in two genes can be explained by either a possible interaction between the encoded proteins (i.e., the formation of a complex) or that they are acting on the same pathway/process. Non-additive interactions between two mutations are frequent and can even be modulated depending on the conditions tested, making it difficult to make genetic predictions. However, overall, the lack of additive effect of the *btpA* and *btpB* mutations is indicative of a functional relationship between these two genes. Whether by compensatory effects along a common pathway or direct molecular interactions is yet to be determined. Interestingly, we did not previously observe an additive effect of both mutations concerning inhibition of innate NFkB nuclear translocation in infected dendritic cells (Salcedo et al 2013).

2. In the discussion, the authors surmise that the lack of effect on endocytosis is cell type specific and further that phagocytes may respond differently. Since both ectopic expression (where endocytosis blocked) and infections were both performed in HeLa cells, I'm not sure I understand this argument. It would seem that the difference in endocytosis effect most likely reflects magnitude of expression. Further, it should be straightforward to infect macrophages with these mutants and determine effects on endocytosis and NAD+ levels.

We have now carried the same experiments in bone marrow-derived macrophages (immortalized), and indeed there is no impact (data included in Figure 7). We have therefore modified the discussion accordingly to suggest that the effect observed in yeast and transfection of HeLa is due to over-expression of BtpA and BtpB (line 509-515).

However, we did observe a definite impact on NAD levels in macrophages and included this data in Figure 10.

3. Timing is a question: what is the time course for NAD+ suppression? Does it take until 48h to see an effect? The Btp proteins are expressed early following infection – could this be because of intracellular Brucella numbers needed to detect an effect?

Indeed, we only observed NAD depletion at 48h post-infection and this is now mentioned in the text (line 396). However, we cannot exclude that this is simply due to the lack of sensitivity of our assay. Due to the high costs of measuring NAD we only performed two experiments at 24h (see Fig.A for reviewers) and instead focused at 48h for which there was a clear phenotype.

Besides, the precise kinetics of secretion of BtpA and BtpB are not well established. We can detect translocated BtpA and BtpB as early as 4h post-infection using the TEM assay (Salcedo et al. 2013) however, it is possible that the peak of translocation is actually at latter stages of the infection (after 24h) or that enough effector needs to accumulate to see an effect. It is also possible that the effectors need to reach a particular intracellular compartment in order to exert their NAD-consuming activity. Inhibition of NFkB was observed at 24h, whereas NAD depletion only at 48h; perhaps there is a sequential series of events taking place or potentially these are two distinct phenotypes. Further work is necessary to define the intracellular localization of BtpA and BtpB during infection. These are challenges we are actively pursuing, but imaging of *Brucella* effectors during infection remains challenging.

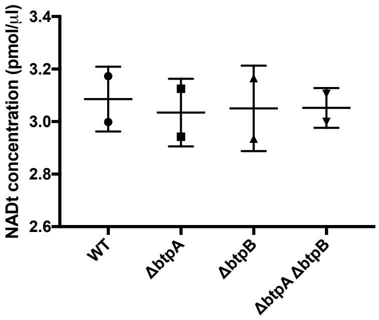

Figure A. Total NAD measured at 24h post-infection in infected HeLa cells (data from 2 independent experiments).

4. Although not necessary, is it possible to detect an effect on glycolysis vs oxidative phosphorylation in mutant infected cells? It would be difficult to tease apart effects on cytokine production because of the other proposed TLR-antagonizing functions of the Btp TIR domains.

This is an excellent suggestion. We are currently awaiting funding to do these experiments while constructing point mutants of *Brucella* for NAD catalytic sites in both *btpA* and *btpB* to address in a more precise manner during infection. Indeed, it is possible that NAD levels may impact this metabolic switch. Furthermore, these effects may also be connected to modulation of the innate immune response (as covered in the discussion) but remains at this stage speculative. We hope to address all these questions in the near future, which we think require another year or two of experiments.

5. Multiple TIR domains have been crystallized. Besides being conserved, what else to these critical residues do? Are they important for folding or TIR-TIR interaction?

We have now integrated a structural analysis of these mutants in Figure 4 and S5 Fig with the help of Laurent Terradot (now an author in the manuscript). Although the structure of BtpB is not yet available, we have found that the mutations isolated in our screen are well conserved in BtpA and other purported NAD^+-hydrolase TIR domains, namely mammalian SARM1 and plant RUN1. We used the BtpA TIR domain structure to map the equivalent residues and this is illustrated in new Fig 4A and S5A-B Fig. Indeed, in the current version of the manuscript, we present and discuss evidence that the mutated residues define structural clues for proper activity and that NADase loss-of-function can be segregated from the ability of BtpA and BtpB TIR domains to assemble filamentous structures.

6. In the yeast, the whole BtpA had no effect on ATP and minimal effect on NAD+, yet during infection, increase in NAD was comparable between BtpA and BtpB mutants. Any thoughts as to why this might be the case?
We discuss in the paper that, contrary to BtpB, the non-TIR N-terminal region of BtpA may exert a negative regulation of the catalytic activity of the TIR C-terminal region. We currently ignore the mechanisms for this negative regulation, which may be relieved during infection by interaction with other translocated effectors or host proteins that are absent in the yeast model. Consistent with the second hypothesis, over-expression of BtpA in human cells did result in a significant reduction of NAD, similarly to BtpB (Fig. 8B).

Part II – Major Issues: Key Experiments Required for Acceptance

Reviewer #2:

1. It is appreciated that a significant amount of work went into producing all of the yeast data (Figures 1-6), but in retrospect what do the investigators feel that the readers can take away from all of the yeast data that has been presented? It appears that most of the observed phenotypes reflect non-specific effects due decrease ATP/NAD levels, presumably a defect that will arise with the expression of many toxic proteins. It does not appear that they would ever have come to the conclusion that BtpA and BtpB might have NAD consuming activity, if not for the prior publication in Current Biology in 2018.
As mentioned above, we first noticed an impact on the ATP levels in yeast when trying to understand how BtpB could be so drastically affecting the yeast cytoskeleton, endocytosis and MAPK. This, together with the results of the suppressor screening, pointed to a specific negative effect on energy metabolism, which is not generally observed when other toxic bacterial effectors are expressed in yeast, in our own experience and that of other groups using this model. This was done, incredibly as it may seem to the reviewer, prior to the groundbreaking paper of Essuman et al in 2018. However, when Essuman et al paper was published, we finally understood the full reason for the activity of the TIR domain and were quite excited about it. Therefore, we went on to expand our study to BtpA and BtpB and also in the context of infection. We are not sure why this is a major issue for the reviewer that could hamper acceptance of the manuscript. We fully acknowledge the Essuman et al work in our introduction as well as throughout the manuscript. Nonetheless, to take into account the comments for the reviewer we have reduced the number of figures describing the initial yeast part from six to four, by moving data related to MAPK signaling from former Fig 2C-F to supplementary (S3 Fig), as well as figures illustrating the screen for yeast suppressors (former Fig 3, now in S4A Fig), fusing figures, and eliminating explanatory schemes.

2. The significance of the yeast overexpressor screen is unclear. Again, it is very unclear how the few hits found in addition to the phenotypes obsereved in yeast would have led the investigators to the discovery that BtpB is a NAD-consuming protein. The hits in this genome-wide screen are quite disparate and are only weak suppressors. It is reassuring that they don't suppress the toxicity observed with overexpression of P13kalpha-CAAX, but this isn't the most convincing argument that their mechanism of suppression is specific to BtpB. Do they also suppress the toxicity associated with expression of the BtpB and BtpA TIR domains? Is it surprising that they did not find overexpressor of proteins involved NAD metabolism that suppress toxicity?
The referee is right about the fact that the hits from the suppressor screen are not directly related to NAD metabolism. However, our observation that some hits were related to the regulation of sugar metabolism, even if their function is not well established in the literature, was a key that led us to test ATP levels before the Essuman et al paper was published. Thus, we would still like to mention the screen in order to present the story as it chronologically happened. Besides, we think that it may be interesting for readers interested in yeast metabolism that overexpression of these genes can partially suppress NAD deficiency in the cell. Fulfilling the referee's demand, we have now tested the suppressors on BtpB and BtpA TIR domains. Peculiarly, partial suppression occurs for BtpB-TIR but not for BtpA-TIR. Thus, this seems rather specific for BtpB-TIR or BtpA-TIR is more robust as a NADase, so these partial suppressors are not strong enough to counteract its toxicity. Considering the reviewer comments, we have moved former Fig 3 together with these new data to Supplementary material and modified the text accordingly.

3. Lines 344-350. This section is confusing. It appears that there is prior evidence that TIR expression stabilizes microtubules, is this also seen here? Or, are the investigators observing that TIR is stabilizing only actin cytoskeleton? It seems like it is the latter. Did the investigators also look at microtubule stability? This experiment is not necessary, particularly as they present no evidence for a role in BtpA or BtpB in altering the status of the cytoskeleton in the context of an infection.

We agree with the reviewer and the data have been removed. The effect on microtubules was described by several other groups for both BtpA and BtpB yet only in ectopic expression and not in infection.

4. In the infection assays, what is the rationale for assaying for changes in NAD levels at the 16 hour time point shown in Fig. 11B? At what time are BtpA and BtpB expressed in intracellular bacteria? When are they observed in the cytosol of host cells? How stable are they? The big question here is whether or not the investigators are studying a direct or indirect effect? Can the investigators complement the btpA and btpB knockouts? Do variants predicted to be catalytically dead no longer complement? The latter are important controls that should be included. Also, it doesn't appear that the actions of BtpA and BtpB are functionally redundant. How do the investigators explain this?

The 16h correspond to the time we use for the cultures of the bacterial inocula, prior to infection. The intracellular NAD measurements were done at 48h post-infection, as at 24h we could not see a difference (see answer to reviewer 3 above and FigA for the reviewers). We have now clarified the text in the manuscript accordingly (line 396).

BtpA and BtpB are detected as translocated into the host cytosol as early as 4h post-infection (Salcedo et al 2013) but it is not clear when their translocation is at its maximum nor how stable they are. We are currently working on imaging these effectors during infection.

As suggested, we attempted to complement the mutants. We could not express BtpA nor BtpB using the Tn7 system we commonly used for our complementation studies. We therefore relied on expression of BtpA and BtpB from a pBBR vector. We made two constructs, with the gene alone and with its own promoter. We observed clear complementation for BtpA in both HeLa and macrophages with both vectors (only the one with the gene alone is included). This complementation was dependent on the catalytic activity as mutations of the catalytic site prevented complementation of BtpA in HeLa cells and BtpA and BtpB in bone marrow-derived macrophages (despite efficient translocation into host cells). Together these results strongly indicate the effects observed on NAD are due to a direct enzymatic activity on NAD and the data are now presented in Figure 9 and 10.

Finally based on Genetics, lack of additive effect of mutations in two genes can be explained by either a possible interaction between the encoded proteins (i.e., the formation of a complex) or that they are acting on the same pathway/process. Non-additive interactions between two mutations (epistatic) are frequent and can even be modulated depending on the conditions tested, making it difficult to make genetic predictions. However, overall, the lack of additive effect of the *btpA* and *btpB* mutations is indicative of a functional relationship between these two genes. Whether by compensatory effects along a common pathway or direct molecular interactions is yet to be determined. Interestingly, we did not previously observe an additive effect of both mutations in relation to inhibition of innate NFkB nuclear translocation in infected dendritic cells (Salcedo et al 2013).

5. In Fig. S6B, are the investigators growing the Brucella under conditions that would induce expression of BtpA and BtpB? If not, the significance of this control is unclear, as presumably the bacteria grow differently in vitro than within the c host cells. Can the investigators, isolate the intracellular Brucella and assess NAD levels? This would be the appropriate control.

We could not unfortunately isolate bacteria from the infected cells and measure the NAD independently. Therefore, this control shows that the differences observed in infected cells are not due to differences in bacterial NAD levels. We have not managed to produce antibodies against BtpB but we know that BtpA is expressed in the wild-type in our growth conditions for the inoculum (see Fig B for reviewers) as are all the effectors we have characterized to date in our lab. However, the home-made antibody we have for BtpA yields many non-specific bands making it impossible to use in infected cells.

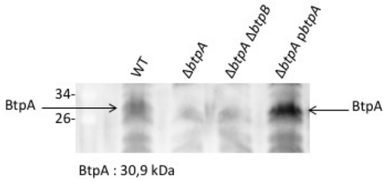

Figure B. Western blot from inocula cultures (16h) probed with an anti-BtpA antibody. Specific band corresponding to BtpA is marked with and arrow and is absent in the mutant strains.

Reviewer #3:

There are no major experimental issues, though performing the experiments requested above in macrophages would significantly enhance impact, particularly as cell type is posited as a reason for differences. Clarification of the timing of NAD+ suppression during infection would be appreciated. Beyond these experiments, the current data requires more explanation.

This experiments have now been carried out and included in the manuscript (Figure 7 and 9). NAD depletion is also observed in macrophages and the phenotype fully complemented. We have also clarified the timing of the NAD experiments in the manuscript.

Part III – Minor Issues: Editorial and Data Presentation Modifications

Please use this section for editorial suggestions as well as relatively minor modifications of existing data that would enhance clarity.

Reviewer #1:

lines 71 and 72 : can we really propose that bacteria have strategies and tactics? I know it is widespread, but I would avoid it

We have modified the sentence accordingly.

line 133 : do the authors really show that there is a blockage of the bioenergetics patways? I agree that a depletion of the pool of NAD+ will have consequences on host metabolism

We have modified the sentence to be precise about the phenotype as well as modified the title

line 171 : these results do not suggest that TIR are prone SELF-interact, they could interact with a filamentous structure in yeast cells

We have modified this statement according to the referee's suggestion: "Although we cannot rule out that *Brucella* TIR domains are interacting with filamentous structures other than tubulin in yeast, our results suggest that they are prone to forming highly ordered structures by self-interaction"

lines 2017-2018 : the western blots with-Kss1 and P-Fus3 are not really convincing in Figures S1A and S1B, are they really necessary?

According to the reviewer's suggestion, these blots have been removed

line 268 : "In agreement", with what?

"In agreement with its lower toxicity". This has been amended.

Fig. S4A and Table S4 : what is the mutation Y187X? did you not have frameshift mutations? or mutations to stop codons? Last column in Table S4 can be deleted (simply state somewhere that they were all isolated once).

Y187X referred to a nonsense mutation generating a stop codon. No frameshift mutations were isolated. It has been noted as Y187stop in the table to avoid confusion. The column mentioned has been deleted.

line 301 : "tolerated" is an interpretation, be more cautious (although I agree that it is likely)

We have modified to sustained.

line 313 : as well as Myc-expressng BtpB?

Modified accordingly.

line 315 : "A proportion", can it be quantified?

We did not quantify the FK2-positive cells as it would not contribute further to our understanding of the function of these effectors, but we have modified the text.

line 376 : I would suggest to move Fig. 11A to the supplementary data

We have decided to keep the endocytosis results in the main paper (together with new data on macrophages) rather than moving it to supplementary as suggested by the reviewer 1 in view of other reviewers' comments. Since no differences were observed in either macrophages and HeLa cells, moving to supplementary would leave the impact on endocytosis following over-expression only and this would in our opinion mislead readers that this was a generalized function of BtpA and BtpB. It is important to show that over-expression can induce phenotypes

not relevant in infection. For consistency with the macrophage experiment we also quantified endocytosis at 24h post-infection for all the strains and replaced the data in Fig S7A.

line 387 : add a reference to show that "it is well established that btp mutants replicate to the same levels". Is it also true for the double deletion strain?
Yes, this is also valid for the double mutant strain using CFU counts. A reference has been added (Salcedo et al 2013).

line 456 : the NAD+ level drops over one order of magnitude, but is it due to the overexpression of the effector?
Indeed we overproduce the heterologous proteins in yeast for functional assessment, often leading to severe effects. Such rough approach drives primary conclusions that only hint the function of the proteins at the finely modulated levels of the infection scenario. In order not to misguide the reader, we have added at the end of this sentence “when overexpressed”..

line 479 : shown to be an important hub?
Modified accordingly.

line 1049 (legend of Fig. 9) : how do you decide that a cell is "showing uptake", is it the case if only one vacuole is positive? or where do you place your cutoff to classify your cells as positive or negative for endocytosis? Please clarify
We have added the following explanation: A cell was considered positive when clear labelling of endocytic vesicles was observed throughout the cell.

Legends of figures 10 and 11 : could you clarify the units used for the evaluation of the NAD+ level? (microliters refer to what?)
The exact calculation is now explained in the methods. The sample volume is taken into account to obtain the total NAD concentration (NAD^+ and NADH).

Reviewer #3:
1. In Fig S1, there are a lot of bands in the gel – how were the relevant ones be identified? Why are the proteins doublets? In this Figure, the Slt2 band is cropped so tightly, it is difficult to see. For the other MAPkinases, the gel has so much spotting, it is difficult to make out bands at all, and a decrease in phosphorylation vs vector control cannot be appreciated. Are there any other blots that could be substituted?
There are multiple degradation bands in the immunoblot with anti-GFP antibodies. This is common upon overexpression in yeast lysates even when appropriate experimental methods are applied and protease inhibitors are added. The lower mobility bands correspond to the full length fusions, according to their molecular weights, and are marked with arrowheads. In the current version of S1A Fig, we have eliminated the Fus3 and Kss1 immunoblots as they were not relevant, as pointed by reviewer 1. We have reduced the cropping for the Slt2 MAPK blots as suggested. Indeed the figure looks nicer. We thank the referee for these suggestions.

2. I could not appreciate the “much larger” E234A aggregates from Figure 8. In S5E, the cell nucleus looks much larger, even though the magnification bar is smaller – there seem to be some other discrepancies between cell size and apparent magnification – is the lowest row of Fig8 at a higher magnification?
Indeed, we had a mistake in the scale bars and apologize; these have now been corrected and the sentenced modified as we should not really infer on the size of the structures without proper quantification.

3. Is the amount of NAD+ significantly increased in the double mutant in Fig. 11? It’s not shown.
No, only statistical differences are shown; all the remaining comparisons are non-significant. The text was modified accordingly.

4. Please provide the references stating that the Btp mutants replicate to the same amount.
Now included in the text; Salcedo et al 2003.

5. For the western blots in Figure 2, could you provide number of times this experiment was done and combine to generate error bars for 2D?
This figure has been moved now to S3B Fig. We maintained the representative immunoblots and provide now in the graph error bars depicting the standard deviation from densitometric data on three different immunoblots performed on individual experiments carried out in different clones. The additional blot images are available upon request for editorial purposes.

6. In Figure 3, what are the “Vectors” vs “vector” row?

Former Fig 3 is now presented as S4A Fig and lettering has been changed to specified the names of the empty vectors used in each row.

7. In Figure 5, it is not clear what the "double mutants" or open red triangles refer to.
This figure is no longer in the paper, as a deeper structural analysis is presented in the current version.
