# Supplementary material for: The TIR-domain containing effectors BtpA and BtpB from Brucella abortus impact NAD metabolism
Source: PLoS Pathog. 2020 Apr 16;16(4):e1007979. doi: 10.1371/journal.ppat.1007979 (PMC7188309; doi:10.1371/journal.ppat.1007979)
Supplement: Attachment — Submitted filename: CoronasSerna and Louche Answers to Referees.docx [file ppat.1007979.s013.docx]

**ANSWERS TO REFEREES**

We thank the reviewers for their positive appreciation.

We agree with reviewer 2 that reorganizing the Figures 5 to 10 in order to decrease emphasis on phenotypes that are not relevant to infection (Fig. 7) and merge related data into single figures (Fig. 8-9-10) might simplify the paper and, perhaps, strengthen the points for the reader. If this is mandatory, we would need an extension of the deadline, as the authors that have the ability to rearrange those figures and fulfil this minor issue are not able to perform that task now due to the COVID-19 outbreak.

**Response to minor issues**

*Reviewer #1: The minor issues that I raised were managed by the authors during revision. I realized that the authors should indicate which strain of B. abortus was used in this study, unless I have missed it in the revised manuscript.*

The strain used is 2308. It has been added to the Materials and Methods Section.

*Reviewer #3: 1. Results question: Sometimes, complementation studies can raise more questions than they solve. In Figure 10, I do not see stats bars between mock infected and wild type - are the wild type NAD levels truly lower than mock? Also, why do deltaBtpB and possibly deltaBtpA have higher NAD+ levels than mock infected? Does this suggest that in the absence of Btp proteins, Brucella might increase NAD, which could limit infection? The reason may not be known, but these results should at least be acknowledged.*

The reason why NAD+ levels are higher in the infected cells than in the mock control is that intracellular NAD+ from the bacteria present in the experiment is added to the count. This is also observed for HeLa cells in Fig. 9. We do not think that this expected artifact deserves to be commented in the text, but we have added to the Legends of Figs. 9 and 10 a comment on this to avoid the confusion noted by the referee.

*2. Method of immortalizing the macrophages should be described or referenced.*

We have added the reference and cited it at the appropriate section of Materials and Methods.

PLOS authors have the option to publish the peer review history of their article (what does this mean?). If published, this will include your full peer review and any attached files.

If you choose “no”, your identity will remain anonymous but your review may still be made public.

Do you want your identity to be public for this peer review? For information about this choice, including consent withdrawal, please see our Privacy Policy.

Reviewer #1: No

Reviewer #2: No

Reviewer #3: Yes: Judith A Smith
